# Safety and efficacy of curcumin versus diclofenac in knee osteoarthritis: a randomized open-label parallel-arm study

**DOI:** 10.1186/s13063-019-3327-2

**Published:** 2019-04-11

**Authors:** Dhaneshwar Shep, Chitra Khanwelkar, Prakashchandra Gade, Satyanand Karad

**Affiliations:** 1 Krishna Institute of Medical Sciences, Satara, Maharashtra India; 2 Dr. Vithalrao Vikhe Patil Foundation’s Medical College & Hospital, Ahmednagar, Maharashtra India; 3 City Care Accident Hospital, Parli Vaijnath, Beed, Maharashtra India

**Keywords:** Knee osteoarthritis, Pain, Curcumin, BCM-95, Anti-flatulent, Weight-lowering, Anti-ulcer

## Abstract

**Background:**

The purpose of this study was to compare the efficacy and safety of curcumin with those of diclofenac in the treatment of knee osteoarthritis (OA).

**Methods:**

In this randomized, open-label, parallel, active controlled clinical study, 139 patients with knee OA were randomly assigned to receive either a curcumin 500-mg (BCM-95^®^) capsule three times daily or a diclofenac 50-mg tablet two times daily for 28 days. Patients underwent assessment at baseline and days 7, 14, and 28. The main outcome measure was severity of pain using visual analogue scale score at days 14 and 28. Knee Injury and Osteoarthritis Outcome Score (KOOS) (at days 14 and 28), anti-flatulent effect (at day 7), anti-ulcer effect, weight-lowering effect, and patient’s and physician’s global assessment of therapy at day 28 were included as secondary outcome measures. Safety after treatment was evaluated by recording adverse events and laboratory investigation.

**Results:**

At days 14 and 28, patients receiving curcumin showed similar improvement in severity of pain and KOOS scale when compared with diclofenac, and the difference was not statistically significant. At day 7, the patients who received curcumin experienced a significantly greater reduction in the number of episodes of flatulence compared with diclofenac (*P* <0.01). At day 28, a weight-lowering effect (*P* <0.01) and anti-ulcer effect (*P* <0.01) of curcumin were observed. None of the patients required H2 blockers in the curcumin group, and 19 patients required H2 blockers in the diclofenac group (0% versus 28%, respectively; *P* <0.01). Adverse effects were significantly less in the curcumin group (13% versus 38% in the diclofenac group; *P* <0.01). Patient’s and physician’s global assessment of therapy was similar in the two treatment groups.

**Conclusion:**

Curcumin has similar efficacy to diclofenac but demonstrated better tolerance among patients with knee OA. Curcumin can be an alternative treatment option in the patients with knee OA who are intolerant to the side effects of non-steroidal anti-inflammatory drugs.

**Trial registration:**

ISRCTN, ISRCTN10074826. Registered 21 November 2017 - Retrospectively registered.

## Background

Knee osteoarthritis (OA) is the fourth leading cause of disability [1]. Symptoms of knee OA normally begin after the age of 40 years but can affect younger people after a traumatic injury. It is highly prevalent among obese patients, and the estimated incidence is 10% to 15% in the population above 60 years of age [1, 2]. Owing to the increasing prevalence of obesity and the aging population, the prevalence of knee OA is expected to increase [3, 4]. Activity restriction and functional limitations among elderly obese patients with knee OA gradually reduce physical, psychological, and social well-being of patients, leading to worsening of their quality of life [5, 6]. Also, it significantly increases the financial burden on patients and families and health-care systems. A study conducted by Dominick et al. (2004) among more than 4000 patients with OA showed worse quality of life in most patients, especially for items related to poor general health, pain, and activity limitation [7].

Pain is one of the key symptoms that drive individuals to seek medical attention and contributes to functional limitations and reduced quality of life [8–11]. Current recommendations for managing OA consider relieving pain, improving physical functions, and slowing the progress of the underlying disease to be important goals of therapy. Usual first-line pharmacologic therapy for knee OA consists of non-steroidal anti-inflammatory drugs (NSAIDs), which provide effective relief of symptoms in most patients [12, 13]. Pharmacological therapy with NSAIDs offers temporary relief in symptoms but is associated with serious risk after long-term use. Chronic administrations of NSAIDs cause gastroduodenal mucosal erosions in about 35–60% of patients and gastric or duodenal ulceration in 10–25% of patients [14]. These events are a consequence of a non-selective mechanism of action of traditional NSAIDs [13]. Additionally, elderly patients are at greater risk for gastrointestinal (GI) bleeding secondary to the use of NSAIDs [15]. Long-term use of non-opioid analgesics such as acetaminophen and NSAIDs, including cyclo-oxygenase-2 (COX-2) inhibitors, has been found to be associated with enhanced risk for GI bleeding, hypertension, congestive heart failure, and renal insufficiency [16]. There is a need for an effective and safer alternative treatment for knee OA.

Curcumin, a polyphenolic compound derived from the dietary spice turmeric (*Curcuma longa*), possesses diverse pharmacologic and biological properties. Curcumin has been used for centuries in traditional Chinese and Ayurvedic medicine for its anti-inflammatory properties [17]. The efficacy of curcumin is shown to be similar to that of ibuprofen for the treatment of knee OA [18]. A study carried out with curcumin on patients with rheumatoid arthritis demonstrated a significant improvement in the duration of walking time and joint swelling which was almost comparable to phenylbutazone [19]. Pre-clinical studies conducted on rats suggested that curcumin is a gastro-protective agent and acts as a potent anti-ulcer compound, protecting against gastric mucosal injury [20, 21]. A study has shown that curcumin acts as a potent anti-ulcer compound to protect indomethacin (NSAID)-induced gastric ulcer [22]. Curcumin inhibits increased acid secretion to prevent ulcer aggravation. Clinical evidence confirmed that curcumin is safe for human use [23, 24]. Anti-flatulent and body weight-lowering effects were also reported with the use of curcumin in pre-clinical studies [25, 26].

However, the poor oral bioavailability of curcumin hampers its therapeutic efficacy, which is a major concern. The bioavailability of curcumin can be increased by combining curcuminoids with essential oil of turmeric [27, 28]. The availability of curcumin in blood plasma was seven times higher after consuming curcuminoids and essential oil of turmeric than normal curcumin. A significant level of curcumin was retained even 8 h after administration and was found to be non-toxic and safe [29–31]. Studies have been conducted on a curcuminoid–essential oil complex, showing that it has therapeutic efficacy in numerous diseases like major depressive disorders, Alzheimer’s disease, and rheumatoid arthritis and has potential for widespread application and radioprotective effect in different kinds of cancer [32–38].

Diclofenac is a well-known NSAID with anti-inflammatory, analgesic, and anti-pyretic properties, comparable or superior to other NSAIDs [39]. Curcumin has been extensively used in traditional medicine in India, particularly as an anti-inflammatory agent [40]. The objective of this study was to compare the efficacy and safety of curcumin with those of diclofenac in patients with knee OA. We also investigated the anti-flatulent and weight-lowering effects of curcumin among patients with knee OA and compared them with those of diclofenac.

## Methods

### Ethics and participant confidentiality

This study was conducted in accordance with the Declaration of Helsinki, the ICH-GCP E6 (R1, R2), and ICMR-National Ethical Guidelines for Biomedical and Health Research 2006. Ethics committee approval was obtained from the Krishna Institute of Medical Sciences, Karad, Maharashtra, India, before initiating the study (reference number: kimsu/PhD/11/2010). Prior to any study-related screening procedures, written informed consent was obtained by the principal investigator from each patient before enrollment in the study. The study was registered with the ISRCTN registry (ISRCTN10074826). Each participant was identified only by the participant study number, and all documents in the study were identified by using the initials and participant study number. The patient identification information was handled only by a delegated staff and stored in locked cabinets accessible only to study staff.

### Trial design and participant selection

This trial was designed as a prospective, randomized, open-label, active controlled parallel-group study. The randomization sequence was generated by an independent statistician using GraphPad Software (GraphPad Software, San Diego, CA, USA) with an allocation ratio of 1:1. Allocation was concealed by using sequentially numbered identical boxes. Patients fulfilling the eligibility criteria were enrolled and randomly assigned to receive either the intervention or the comparator. The pharmacist designated by the investigator dispensed the investigational products.

This study was conducted at City Care Accident Hospital, Parli Vaijnath, Maharashtra, India. All patients (ages 38–65 years) with symptomatic knee OA for at least 3 months with no joint deformities and requiring treatment with anti-inflammatory drugs were screened for eligibility after providing written informed consent. Patients meeting the American College of Rheumatology (ACR) criteria for knee OA (confirmed by x-ray) and having moderate pain (visual analogue scale (VAS) score of 4 or greater) in the knee joint were included in the study. Patients taking analgesics were given a washout period of at least 3–7 days (or longer depending on the pharmacokinetic of drug) before starting the study intervention. The dietary intake of curcumin was restricted. The patients were advised not to change their routine dietary habits and physical activity which can lead to weight gain or loss. Patients with flatulence episodes ranging from 5 to 20 per day were enrolled to evaluate anti-flatulent effects.

The following patients were excluded from the study: those who received a corticosteroid injection within the previous 4 weeks; had a history of active peptic ulcer, gastric ulceration, stomach pain, or GI bleeding or bleeding disorders; had secondary OA due to syphilis, metabolic bone disorder, or acute trauma; required prescription anticoagulants, hydantoin, lithium, steroids, methotrexate, and colchicines or concurrent pain-relieving medication such as tranquilizers, hypnotics, excessive alcohol, or any other drug affecting the evaluation of analgesic action; or had known hypersensitivity to diclofenac sodium and turmeric. Patients with a medical history of significant impairment of hepatic or renal functions, cardiac insufficiency, and bronchitis were also excluded. Pregnant and lactating women and women of child-bearing age not using or not willing to use contraceptives were not included.

### Interventions and dosage

The intervention used in this clinical trial was curcumin (BCM-95^®^) 500-mg zero size hard gelatin capsule (Curcugreen^®^, Arjuna Natural Ltd., Kerala, India). Each capsule contained curcuminoids and essential oil of turmeric complex (curcumin, demethoxycurcumin, bisdemethoxycurcumin, and volatile oils from turmeric rhizome) total not less than 95%, curcuminoids not less than 88%, and curcumin not less than 68%. The comparator used was diclofenac 50 mg uncoated tablet (Lupin Pharmaceuticals, Mumbai, India).

The dosage was curcumin 500 mg three times daily or diclofenac 50 mg two times daily for 28 days. Patients were also provided with rescue medications paracetamol 500-mg tablet (Calpol, GlaxoSmithKline Pharmaceuticals Ltd., Mumbai, India) and ranitidine 150-mg tablet (Rantac, J. B. Chemicals and Pharmaceuticals Ltd., Mumbai, India).

### Assessments

Patients were evaluated at baseline (day 0) and week 2 (day 14) and week 4 (day 28). Improvement in pain intensity on the VAS at each evaluation visit was considered the primary outcome. The VAS is a valid assessment tool. It brought greater sensitivity and greater statistical power to data collection and analysis by allowing a broader range of responses. It removed bias that was introduced by examiner questioning, and it allowed graphic temporal comparisons [41]. Secondary outcomes were improvement in the pain intensity on Knee Injury and Osteoarthritis Outcome Score (KOOS) subscale, anti-flatulent effect, weight-lowering effect, patient’s global assessment for overall symptom relief, physician’s global evaluation of treatment, and anti-ulcer effect. The KOOS is a valid, reliable, and responsive self-administered instrument used for evaluating short-term and long-term follow-up of knee injury, including OA [42]. The patients were asked to record the number of episodes of flatulence over the previous 24 h before administering medication and on day 7. The anti-flatulent effect was assessed by comparing the mean fall in number of episodes of flatulence. The weight-lowering effect was assessed by comparing the mean fall in weight on day 28 with baseline weight. Anti-ulcer effect was assessed by recording the number of H2 blocker tablets that patients consumed during the study period. The requirement of rescue medication throughout the study period was recorded in a case report form. Compliance with study medications was checked in each visit. Adverse events (AEs) reported or observed during the study period were recorded in the case report form at each study visit.

### Statistical analysis

Sample size calculation was performed by using PS: Power and Sample Size Calculations software (version 3). Based on a power of 80% and a type I error rate of alpha of 0.05 (two-tailed), a sample size of 65 participants per group was required to detect an estimated difference of 1.24 in the mean pain scores between the treatment arms with a standard deviation of 2.5. Given a dropout rate of 5%, a total sample size of 69 participants per treatment group was considered sufficient in this study. All statistical analyses were performed on an intention-to-treat basis with the last observation carried forward method. Normality was tested for the data by using the Shapiro–Wilk test. The paired *t* test or unpaired *t* test was used for normally distributed data and the Wilcoxon signed-rank test or Mann–Whitney test was used for the non-normally distributed data to compare the continuous data within groups and between groups, respectively, and the chi-squared or Fisher’s exact test was used to compare the categorical data of study groups. For comparison of VAS score between the groups, the independent *t* test was used as the scores were not on one of the extremes. A comparison of two treatments (curcumin and diclofenac) with the perfect analgesic was also carried out, and the correlation coefficient was determined. A *P* value of less than 0.05 was considered statistically significant. All statistical analyses were performed by using SPSS version 24.

## Results

### Patient disposition and characteristics

**Fig. 1:**
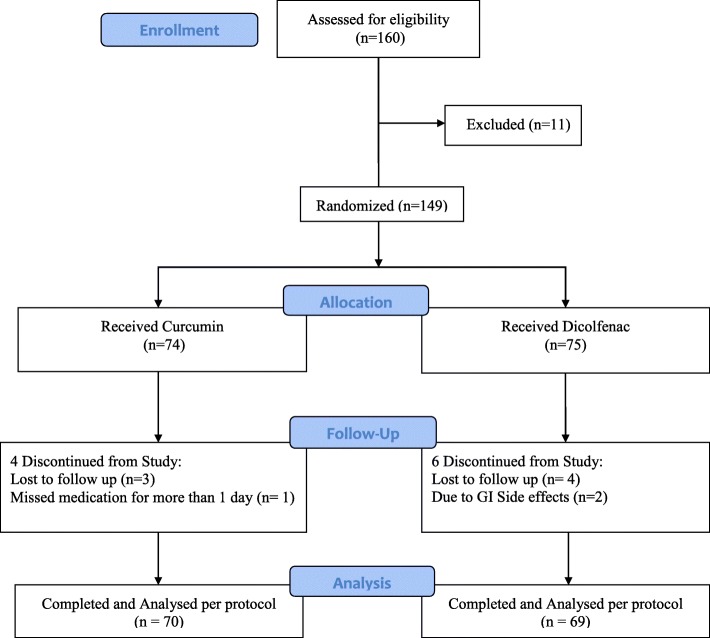
Flow diagram of participants

One hundred sixty patients were screened and 149 patients were enrolled in the study. A total of 139 patients (70 in the curcumin group and 69 in the diclofenac group) completed the study and were subjected to statistical analysis. Dropouts included four patients from the curcumin group and six from the diclofenac group (Fig. 1). The treatment groups were comparable in terms of demographic characteristics (i.e., age, weight, height, and gender). Clinical assessment of pain on VAS and KOOS subscale at the start of the trial (baseline) was similar between the treatment groups. Overall, demographic and baseline characteristics between the treatment groups were similar before the start of study treatment (Table 1).

**Table 1:** Demographic and baseline characteristics in patients with knee osteoarthritis

Patient characteristic	Curcumin (*N* = 70)	Diclofenac (*N* = 69)
Age, years	53.09 ± 4.17	52.14 ± 3.76
Gender, male/female	45/25	48/21
Weight, kg	61.37 ± 5.46	63.51 ± 5.19
Duration of knee osteoarthritis, months	7.40 ± 3.53	7.45 ± 3.15
Baseline pain intensity on visual analogue scale	7.84 ± 0.63	7.81 ± 0.73

Values are expressed in mean ± standard deviation except for gender variable (presented as number of patients in each category). Visual analogue scale is from 0 to 10, where 0 indicates “No pain” and 10 indicates “Worst possible pain”

### Efficacy results

Both treatment groups showed a significant reduction in VAS scores on day 28 from their baselines (*P* <0.01). However, there was no significant difference in reduction in pain intensity between the groups. The numbers of patients having more than 50% improvement in VAS score were 66 in the curcumin group and 67 in the diclofenac group. This difference was not statistically significant (*P* = 0.68) (Table 2).

**Table 2:** Comparison of pain as determined by visual analogue scale in patients with knee osteoarthritis

Visit	Curcumin (*N* = 70)	Diclofenac (*N* = 69)	*P* value
Baseline	7.84 ± 0.63	7.81 ± 0.73	0.79^t^
Day 14	4.69 ± 0.79	4.58 ± 0.60	0.38^t^
Day 28	2.20 ± 0.81	2.20 ± 0.61	0.98^t^
Change at day 14	−3.16 ± 0.79	−3.23 ± 0.91	0.61^t^
Change at day 28	−5.93 ± 0.99	−5.61 ± 0.88	0.82^t^
*P* value	*P* <0.01^wc^	*P* <0.01^wc^	
VAS reduction % ≤ 50	*N* = 4	*N* = 2	*P* = 0.68^f^
VAS reduction % > 50	*N* = 66	*N* = 67	

Values are expressed in mean ± standard deviation

*Abbreviations*: *f* Fisher exact test, *N* number of patients in each group, *t* independent t test, *wc* Wilcoxon signed-rank test

*P* <0.05 is considered a statistically significant difference. Visual analogue scale (VAS) is from 0 to 10, where 0 indicates “No pain” and 10 indicates “Worst possible pain”. Change in mean score at days 14 and 28 is calculated from baseline VAS score

**Fig. 2:**
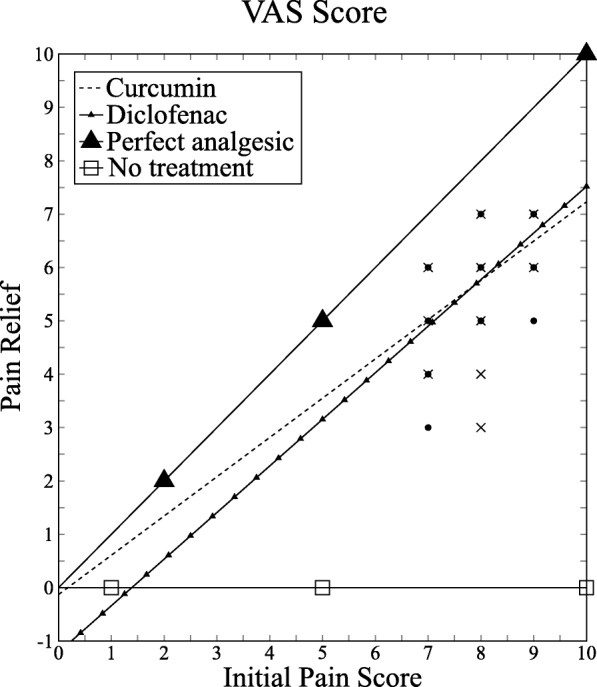
Visual analogue scale (VAS) plot: Relationship between pain relief measured by subtraction and initial pain score for curcumin and diclofenac. Correlation coefficients: curcumin, 0.5; diclofenac, 0.73

In our study, pain relief for diclofenac and curcumin are well below the “perfect analgesic” (slope = 1) and above “no treatment” (slope = 0) graph, and the contribution of slope change due to the initial pain score is nil and statistically not significant (*P* = 0.79) (Fig. 2).

Both treatment groups showed continuous improvement in scores in all five subscales of KOOS at each treatment visit from baseline, and the difference at the end of the study was statistically significant (*P* <0.01). Patients receiving curcumin showed similar improvement in KOOSs in all subscales as compared with diclofenac at the end of study, and the difference was not statistically significant (Table 3).

**Table 3:** Assessment of Knee Injury and Osteoarthritis Outcome Score subscale in patients with knee osteoarthritis

KOOS subscale*	Visit	Curcumin (*N* = 70)	Diclofenac (*N* = 69)	*P* value^m^
Pain	Baseline	53.29 ± 5.70	53.15 ± 4.24	0.89
	Day 14	72.70 ± 4.89	73.69 ± 3.97	0.56
	Day 28	88.77 ± 5.62	90.38 ± 3.61	0.07
	Change at day 14	19.40 ± 2.89	20.55 ± 3.22	0.25
	Change at day 28	35.48 ± 4.07	37.23 ± 2.05	0.15
	*P* value^wc^	<0.01	<0.01	
Symptoms	Baseline	55.77 ± 6.00	55.87 ± 5.97	0.66
	Day 14	61.58 ± 5.65	61.93 ± 5.47	0.88
	Day 28	67.95 ± 5.86	69.13 ± 5.50	0.82
	Change at day 14	5.82 ± 3.81	6.06 ± 3.36	0.83
	Change at day 28	12.19 ± 5.28	13.26 ± 4.44	0.94
	*P* value^wc^	<0.01	<0.01	
Function in daily living	Baseline	58.45 ± 4.96	61.26 ± 5.24	0.21
	Day 14	77.67 ± 5.90	80.54 ± 6.10	0.05
	Day 28	94.58 ± 6.58	96.23 ± 2.71	0.49
	Change at day 14	19.22 ± 2.99	19.28 ± 2.38	0.55
	Change at day 28	36.13 ± 3.61	34.97 ± 5.05	0.34
	*P* value^wc^	<0.01	<0.01	
Function in sport and recreation	Baseline	44.86 ± 8.47	48.55 ± 7.58	<0.01
	Day 14	68.43 ± 5.81	71.88 ± 5.01	<0.01
	Day 28	89.14 ± 6.43	91.88 ± 4.38	<0.01
	Change at day 14	23.57 ± 4.27	23.33 ± 4.34	0.63
	Change at day 28	44.28 ± 6.44	43.33 ± 6.22	0.15
	*P* value^wc^	<0.01	<0.01	
Quality of life	Baseline	42.95 ± 5.31	41.91 ± 6.88	0.06
	Day 14	59.64 ± 6.08	59.15 ± 7.73	0.58
	Day 28	73.57 ± 7.46	72.26 ± 8.61	0.64
	Change at day 14	16.70 ± 8.22	17.23 ± 8.44	0.62
	Change at day 28	30.63 ± 10.16	30.35 ± 10.02	0.60
	*P* value^wc^	<0.01	<0.01	

*Higher score indicated better improvement.

Values are expressed in mean ± standard deviation

*Abbreviations*: *KOOS* Knee Injury and Osteoarthritis Outcome Score, *m* Mann–Whitney test (between the group), *N* number of patients in each group, *wc* Wilcoxon signed-rank test (within the group)

*P* <0.05 is considered a statistically significant difference. KOOS scale is from 0 to 100, where 0 indicates “Extreme Problem” and 100 indicate “No Problem”. Change in mean score at days 14 and 28 is calculated from baseline KOOS for each subscale

The numbers of episodes of flatulence between the two treatment groups at baseline were comparable. Patients who received curcumin experienced a significantly greater reduction in the number of episodes of flatulence from baseline compared with patients who received diclofenac (*P* <0.01) (Table 4). The patients who received curcumin experienced a significantly greater reduction in body weight from baseline (*P* <0.01) compared with patients who received diclofenac (*P* = 0.22). The differences in anti-flatulent effect and weight loss in patients between the groups were statistically significant (*P* <0.01) (Table 4).

**Table 4:** Comparison of anti-flatulent and weight-lowering activity

Visit	Curcumin (*N* = 70)	Diclofenac (*N* = 69)	*P* value^m^
Anti-flatulent activity	*N* = 25	*N* = 25	–
At baseline	12.04 ± 2.17	11.32 ± 2.21	0.29
Day 7	2.32 ± 1.11	10.8 ± 2.1	<0.01
Weight-lowering activity	*N* = 70	*N* = 69	–
At baseline	61.37 ± 5.46	63.51 ± 5.19	0.02
Day 28	60.36 ± 5.19	63.39 ± 5.21	<0.01
Change from baseline (at day 28)	1.01 ± 0.81	0.12 ± 0.70	<0.01
*P* value of weight-lowering activity^wc^	*P* <0.01	*P* = 0.22	

Values are expressed in mean ± standard deviation. Data were analyzed by Wilcoxon signed-rank test (wc) and Mann–Whitney test (m). N = number of patients in each group. *P* <0.05 is considered a statistically significant difference

Global assessments of treatment by patient and physician on the basis of overall efficacy and safety were similar for the two treatments (curcumin and diclofenac) (Table 5). The need for rescue medication (paracetamol) was numerically higher in the curcumin group (15 patients; 21%) compared with the diclofenac group (12 patients; 17%) but the difference was not statistically significant (*P* = 0.67). None of the patients required H2 blockers in the curcumin group when compared with the diclofenac group (0% versus 28%, respectively; *P* <0.01) and this indicates an anti-ulcer effect of curcumin.

**Table 5:** Global assessment by physicians and patients after study drug treatment

Global assessment rating	Physician’s global assessment		Patient’s global assessment	
	Curcumin (*N* = 70) n (%)	Diclofenac (*N* = 69) n (%)	Curcumin (*N* = 70) n (%)	Diclofenac (*N* = 69) n (%)
Excellent	10 (14)	10 (14)	11 (16)	12 (17)
Good	56 (80)	54 (78)	54 (77)	52 (75)
Fair	1 (1)	1 (1)	2 (3)	1 (1)
Poor	3 (4)	4 (6)	3 (4)	4 (6)
*P* value	>0.05		>0.05	

Values are expressed as absolute number of patients (percentage) in each category. Data were analyzed by chi-squared test. *P* <0.05 is considered a statistically significant difference. *Abbreviations*: *n* number of patients in each category, *N* total number of patients in each treatment group

### Safety variables

Overall, 13% of patients receiving curcumin and 38% of patients receiving diclofenac reported at least one AE and this difference was statistically significant (*P* <0.01). All reported AEs were mild and transient. The most common AEs were nausea, diarrhea, abdominal pain/acidity, and flatulence. The incidence of each AE was significantly less in the curcumin group compared with the diclofenac group. The relative risk of all AEs except nausea and diarrhea in the curcumin group was reduced to less than 10%, which was clinically significant. The relative risk of nausea and diarrhea was reduced to only 80% and 60%, respectively. Relative risk was calculated by comparing the curcumin group and the diclofenac group (Table 6). Analysis of laboratory values did not reveal any significant adverse outcomes (Table 7).

**Table 6:** Summary of adverse reactions in each treatment group

Adverse reactions	Curcumin^a^ (*N* = 70)	Diclofenac^b^ (*N* = 69)	RR	RRLB	RRUB	NNT
	n (%)	n (%)				
Total number of patients experiencing AEs*	9 (13%)	26 (38%)				
Dyspepsia	0	6 (8.7%)	0.08**	0	1.3	12
Nausea	6 (9%)	7 (10.14%)	0.8	0.3	2.4	64
Vomiting	0	7 (10.14%)	0.07**	0	1.1	10
Diarrhea	5 (7%)	8 (11.6%)	0.6	0.2	1.8	23
Constipation	0	6 (8.7%)	0.08**	0	1.3	12
Abdominal pain/acidity	0	19 (27.53%)	0.03**	0	0.4***	4
Flatulence	0	9 (13.04%)	0.05**	0	0.9***	8
Upper respiratory tract infection	0	5 (7.25%)	0.09**	0	1.6	14

Values are expressed as absolute number of patients (percentage) in each category

*Abbreviations*: *a* treatment group, *b* control group, *n* number of patients in each category, *N* total number of patients in each treatment group, *NNT* number needed to treat, *RR* relative risk, *RRLB* relative risk lower boundary, *RRUB* relative risk upper boundary

*P <0.01 for curcumin versus diclofenac

**Clinically significant adverse event (AE) (RR <0.5)

***Statistically significant AE (95% confidence interval does not include 1)

**Table 7:** Laboratory-based evaluations of safety

Laboratory parameter	Curcumin (*N* = 70)			Diclofenac (*N* = 69)			*P* value^#^
	Before treatment	After treatment	*P* value*	Before treatment	After treatment	*P* value*	
Hemoglobin, gm/dL	14.67 ± 0.37	14.67 ± 0.36	0.29	14.67 ± 0.35	14.74 ± 0.37	0.03	0.37
Red blood cell count, million/mm^3^	5.01 ± 0.32	5.06 ± 0.31	0.06	5.06 ± 0.34	5.12 ± 0.36	0.02	0.29
Total white blood cell count, /mm^3^	7577.14 ± 1723.26	7841.43 ± 1512.60	0.14	7633.33 ± 1841.09	7682.61 ± 1603.76	0.70	0.59
Red cell absolute values							
Packed cell volume %	44.79 ± 2.72	45.06 ± 2.51	0.51	44.86 ± 2.88	45.38 ± 2.77	< 0.01	0.40
Mean corpuscular volume, cubic microns	80.56 ± 3.13	80.91 ± 2.91	0.14	81.20 ± 3.47	81.50 ± 3.45	0.06	0.46
Mean corpuscular hemoglobin, picograms	29.02 ± 1.34	29.23 ± 1.23	0.06	29.17 ± 1.52	29.29 ± 1.39	0.17	0.82
Mean corpuscular hemoglobin concentration, g/dL	33.62 ± 0.89	33.66 ± 0.82	0.69	33.68 ± 0.97	33.74 ± 0.97	0.15	0.42
Differential count							
Neutrophils %	48.67 ± 4.84	49.08 ± 4.72	0.58	48.85 ± 4.45	49.81 ± 4.68	0.03	0.43
Lymphocytes %	28.58 ± 5.24	28.99 ± 5.09	0.50	28.30 ± 4.74	29.19 ± 4.86	0.06	0.80
Eosinophil %	3.57 ± 0.58	3.39 ± 0.55	0.10	3.61 ± 0.57	3.52 ± 0.58	0.48	0.15
Monocytes %	3.96 ± 0.91	4.06 ± 0.80	0.50	4.03 ± 0.74	4.09 ± 0.80	0.70	0.81
Basophils %	0	0	–	0	0	–	–
Peripheral smear examination							
Platelets, /mm^3^	269,757.1 ± 55,561.02	268,514.3 ± 57,049.62	0.72	286,942 ± 49,707.34	279,956.5 ± 60,135.25	0.37	0.30
Erythrocyte sedimentation rate, /hr	13.06 ± 2.90	12.86 ± 2.59	0.59	12.88 ± 2.83	12.93 ± 2.82	0.78	0.82
Biochemistry							
Serum creatinine, mg/dL	0.88 ± 0.27	0.91 ± 0.26	0.36	0.93 ± 0.30	0.96 ± 0.30	0.05	0.41
Serum glutamic pyruvic transaminase (SGPT), IU/L	22.71 ± 5.26	23.6 ± 4.75	0.42	25 ± 4.81	23.71 ± 5.45	0.13	0.98
Serum glutamic oxaloacetic transaminase (SGOT), IU/L	23.6 ± 4.17	23.79 ± 3.51	0.76^t^	25.12 ± 4.19	24.15 ± 4.04	0.12	0.71

Data are expressed as mean ± standard deviation

*P value (within the group) by Wilcoxon signed-rank test

^#^*P* value (between the groups) (after treatment) by Mann–Whitney test

^t^Paired t test

## Discussion

This study demonstrated that curcumin has a similar pain relief effect on patients with knee OA compared with diclofenac. For KOOS subscales of symptoms, functions in daily living, functions in sports and recreation, and knee-related quality of life, curcumin showed improvement comparable to that of diclofenac. Overall, curcumin showed similar improvements in pain, stiffness, symptoms, functions of daily living, sports or recreational activities, and quality of life that have been attributed to its ability to inhibit COX-2, which results in the suppression of prostaglandin synthesis. Furthermore, curcumin has been shown to suppress several pro-inflammatory cytokines and mediators of their release, such as tumor necrosis factor-alpha (TNF-α), interleukin 1 (IL-1), IL-8, and nitric oxide synthase.

Poor bioavailability is the major drawback of free curcumin. In the meta-analysis of data from clinical trials with curcumin supplementation, it was reported that reduction in pain severity did not reach statistical significance with respect to treatment duration. The results of subgroup analysis confirmed that bioavailable optimized preparations increased the analgesic effect of curcuminoids [43]. Beneficial results obtained in our study are possibly due to the combination of curcuminoids and essential oil of turmeric present in curcumin capsules which increased the bioavailability.

Permeability of curcumin is increased by turmerones, which act by inhibiting p-glycoprotein [44]. Inflammation-associated colon carcinogenesis was prevented by the synergistic combination of curcumin and turmerones [45]. The superiority of curcuminoids with essential oil of turmeric with turmerones was reported in many research papers. The anti-inflammatory and synergic potential of curcuminoid essential oil of turmeric complex showed superior protection from dextran sodium sulfate (DSS)-induced colitis in comparison with curcumin alone [46]. A study carried out on the same composition (BCM-95^®^) with *Boswellia serrata* reduced pain-related symptoms in patients with OA and was effective in treating knee OA in comparison with celecoxib (NSAID) [47, 48]. Research on the same composition showed significantly better results in active rheumatoid arthritis when compared with diclofenac sodium [35].

Weight loss can be considered beneficial in the treatment of knee OA; it has been reported that weight reduction of 10% results in improved function by 28% in patients with knee OA [49]. In the study, significant body weight reduction from baseline was shown in the patients who received curcumin (1.65% weight reduction in the curcumin group and 0.19% weight reduction in the diclofenac group). A similar effect from curcumin was reported by Ejaz et al. [26]. Curcumin suppresses angiogenesis in adipose tissue together with its effect on lipid metabolism in adipocytes may contribute to lower body fat and body weight gain. Curcumin induces apoptosis by suppressing the differentiation of preadipocytes to adipocytes. Adipokine-induced angiogenesis of human endothelial cells is also inhibited by suppressing the expression of vascular endothelial growth factor-alpha. Curcumin increases the activation of AMP-activated protein kinase (AMPK) in adipocytes. Phosphorylation of the alpha-subunit of AMPK and suppressing the expression of aminocyclopropane carboxylic acid activate AMPK in adipocytes. A beneficial effect of curcumin in obesity is obtained by increasing the fatty acid oxidation in adipocytes [50]. Hence, the weight reduction caused by curcumin can be beneficial in the treatment of knee OA, especially in obese patients.

In our study, the patients who received curcumin experienced fewer GI-related side effects compared with patients who received diclofenac. Fewer GI-related side effects may be due to potent anti-ulcer action or protection against gastric mucosal injury by curcumin. The mechanism of anti-ulcer activity of curcumin is also well understood. Neutrophils, lymphocytes, and monocytes/macrophages at the inflammatory site in the stomach are activated primarily by the local inflammatory cytokine IL­6. This initiates different oxidative bursts of toxic metabolites and lysosomal enzymes which are responsible for local tissue damage in peptic ulcer disease. The severity and duration of inflammation, particularly in its acute phase, can be predicted more precisely by the pro-inflammatory IL­6 than TNF­α. Curcumin exhibits its anti-ulcer activity by inhibiting IL­6 secretion and also by affecting oxidative stress by its total antioxidant capacity [51]. According to another study, curcumin protects gastric damage by efficient removal of H_2_O_2_ and H_2_O_2_-derived sulfenic acid (SOH) by preventing peroxidase inactivation by NSAID [22]. There is also evidence of possible involvement of glutathione in the curcumin-mediated gastro-protection [21].

Patients who received curcumin experienced a significantly greater reduction in the number of episodes of flatulence from baseline compared with patients who received diclofenac. The effect of curcumin on intestinal gas formation has been demonstrated in *in vitro* and *in vivo* experiments [52]. Thus, the reduction in the number of episodes of flatulence may be associated with the anti-flatulent effect of curcumin.

The VAS is most commonly used to assess pain intensity. It is reliable, valid, sensitive to change, and easy to administer for measuring pain severity [53]. In clinical trials, measuring pain relief is preferred to pain severity as it is not dependent on initial pain severity, equality of the change in different parts of the scale, and variation in the patient’s expression. The effectiveness of any particular treatment can be accurately measured by pain relief, which is the change between pain score after treatment and initial pain score [54]. As long as all data points are in between “no treatment” and “perfect analgesic”, a steeper line is directly proportional to better treatment efficacy. The effect of the slope variation and its effect on the misinterpretation of the results have already been reported [55]. A significant difference in the initial pain score can misguide the interpretation of the result. In our study, there is no chance for misinterpretation of the results. Pain relief for diclofenac and curcumin are well below the “perfect analgesic” (slope = 1) and above “no treatment” (slope = 0) graph. The contribution of slope change due to the initial pain score is nil and statistically not significant (*P* = 0.79). The slope variation obtained in the study results indicates the treatment effect of both groups.

In estimations of pain relief, it may not be appropriate simply to compare only the scores before and after treatment, because the magnitude of this difference is limited by the placement of the initial mark [56]. The quantal method measures pain relief on the basis of the proportion of patients achieving a defined degree of pain relief. Such a method is not suitable for testing drugs which produce moderate pain relief, and a more sensitive method is required. Pain severity assessment based on the percentage change from the initial level and a minimum cutoff of 50% in pain relief greatly improves the sensitivity of pain measurement scales [55]. In our study, the curcumin and diclofenac groups had almost equal proportions (*n* = 66 and *n* = 67, respectively) of patients who had more than a 50% reduction in VAS scores from baseline levels with a *P* value of 0.68.

Significantly fewer patients in the curcumin group reported AEs compared with the diclofenac group. None of the patients in the curcumin group required H2 blockers. The favorable safety profile in the curcumin group was observed because of its gastro-protective and anti-ulcer effect, which may be an alternative to the GI side effect of NSAIDs. Overall assessment of efficacy and safety of treatment by patient and physician was similar for the two groups.

## Limitations of the study

The open-label study design without a placebo-controlled group was one of the limitations of the study. The treatment duration of 28 days may not be sufficient to assess long-term efficacy and prevention of progression of disease as evidenced by structural damage in patients with OA. Hence, a long-term study is warranted with curcumin in patients with OA. Although validated scales were used, the efficacy of curcumin in the treatment of OA was not based on objective measures but on subjective measurement of pain, which is another limitation of the study.

## Conclusions

Our findings suggest that curcumin three times daily has similar efficacy to but a better safety profile than diclofenac two times daily among patients with knee OA. Our study results suggest that curcumin with increased bioavailability (BCM-95^®^) can be a good alternative treatment option in patients with knee OA who are intolerant to the side effects of NSAIDs.

## Abbreviations

AE: Adverse event
AMPK: AMP-activated protein kinase
COX-2: Cyclo-oxygenase-2
GI: Gastrointestinal
IL: Interleukin
KOOS: Knee Injury and Osteoarthritis Outcome Score
NSAID: Non-steroidal anti-inflammatory drug
OA: Osteoarthritis
TNF­α: Tumor necrosis factor-alpha
VAS: Visual analogue scale
